# Management of skin sarcoidosis with minocycline monotherapy

**DOI:** 10.1002/rcr2.413

**Published:** 2019-03-13

**Authors:** Shinichi Sasaki, Motoyasu Kato, Kota Nakamura, Yukiko Namba, Osamu Nagashima, Kazuhisa Takahashi

**Affiliations:** ^1^ Department of Respiratory Medicine Juntendo University Urayasu Hospital Chiba Japan; ^2^ Department of Respiratory Medicine Juntendo University, Graduate School of Medicine Tokyo Japan

**Keywords:** Minocycline, monotherapy, Propionibacterium acnes, sarcoidosis, skin

## Abstract

A 46‐year‐old woman with severe skin sarcoidosis, mainly on the back of the trunk, persisting for >15 years, was followed up without systemic treatment. In 2014, she was started on minocycline monotherapy owing to worsening of the skin sarcoid lesions. Surprisingly, after approximately 1 year of the monotherapy, nearly all skin lesions resolved with only light residual scars, despite the poor efficacy of the monotherapy for pulmonary sarcoidosis. The patient's serum angiotensin‐converting enzyme levels also decreased to the normal range. The presence of Propionibacterium acnes was confirmed when a retrospectively immunostained epithelioid granuloma, obtained from skin biopsy, demonstrated staining with monoclonal antibodies specific for *P. acnes*. Minocycline monotherapy, thus, appears to be a possible treatment modality for skin sarcoidosis.

## Introduction

Sarcoidosis is a systemic granulomatous disease of unknown origin; it leads to the formation of epithelioid granulomas without caseous necrosis in systemic organs and has various clinical symptoms. Although spontaneous remission occurs in many cases, the disease becomes intractable because of progression to the chronic state or repeated recurrence, which reportedly occurs in approximately 30% of patients. A common treatment strategy is to use immunosuppressive therapy, primarily corticosteroids. However, the long‐term administration of corticosteroids causes many adverse events, which may also affect the therapy outcome. Although the cause of sarcoidosis remains unknown, *Propionibacterium acnes*, the only microorganism to be isolated and cultured from sarcoidosis lesions, has garnered attention as the most likely pathogenic bacterium 1. Various antimicrobial agents have been used to eradicate *P. acnes*; particularly, minocycline is the most frequently used antimicrobial agent and has recently been reported to be effective for treating skin sarcoidosis 2. We present a case in which the use of minocycline monotherapy resulted in the complete remission of skin sarcoidosis that had remained untreated without remission for 15 years.

## Case Report

A 46‐year‐old woman with sarcoidosis was followed up at Juntendo Urayasu Hospital from May 1993 onward. Her chief complaint was visual disturbance due to uveitis, and steroid‐containing eye drops were administered. On 14 March 1994, she visited the hospital complaining of sudden right visual disturbance along with a severe headache and was diagnosed with vitreous haemorrhage. She was admitted to our hospital for surgery on 9 May and started on oral steroid therapy with methylprednisolone (20 mg). On 12 May, she underwent vitrectomy combined with endoscopic photocoagulation surgery for her right eye, and the corticosteroid dose was gradually tapered off before being discontinued.

**Figure 1:**
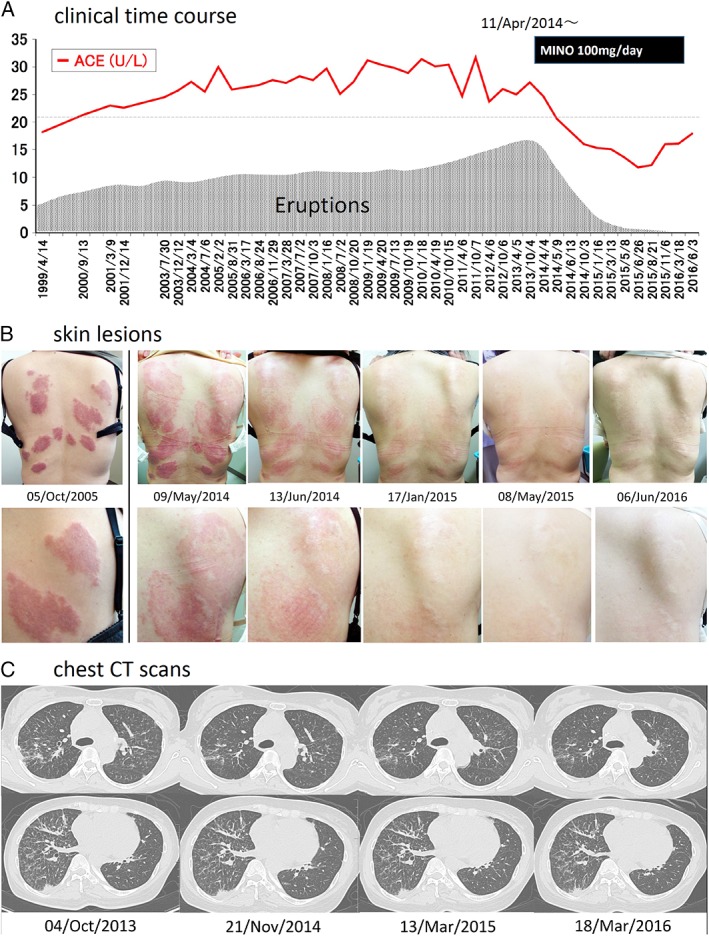
(A) Clinical time course. Oral administration of minocycline was started at 100 mg/day on 11 April 2014. After treatment initiation, the angiotensin‐converting enzyme levels, which had remained high since 2000, rapidly decreased to the normal range. (B) Time course of skin sarcoidosis. Since the early stage of the disease, multiple red sarcoid eruptions appeared mainly on the skin of the back. After minocycline monotherapy initiation, these eruptions gradually resolved and only slight scars remained after approximately 1 year of therapy. (C) Chest computed tomography (CT). High‐resolution chest computed tomography revealed small nodular lesions, reticular lesions, and consolidations along the interstitium, including the bronchovascular bundle or interlobular septa. Although some lesions resolved after the start of minocycline monotherapy, improvement in these lesions was less as compared with those for skin eruptions.

**Figure 2:**
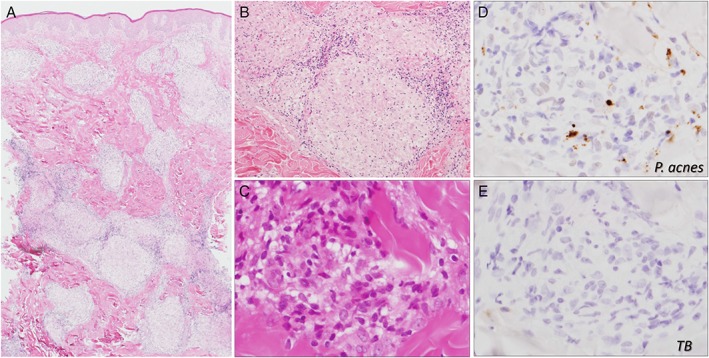
Skin biopsy. (A–C) Multiple non‐caseating epithelioid granulomas can be observed under the epidermis. (D) Anti‐Propionibacterium acnes antibody (PAB antibody*) staining revealed granules of *P. acnes* in the epithelioid granulomas. (E) No staining was observed with the anti‐TB antibody**, that is, the control antibody. *PAB antibody: *P. acnes‐*specific monoclonal antibodies that react with cell‐membrane‐bound lipoteichoic acid. **TB antibody: anti‐*Mycobacterium tuberculosis* antibody.

After the surgery, she was followed up without the oral steroid therapy. In early 1998, she developed some skin eruptions (resembling raised erythematous smooth plaques) on her back that continued to gradually progress (Fig. 1B). Skin biopsy performed on 14 April 1999 revealed multiple granulomatous lesions, confirming a diagnosis of sarcoidosis (Fig. 2D). At that time, a chest computed tomography (CT) revealed diffused small nodules and consolidations in her lung field, indicating a pulmonary involvement of sarcoidosis. However, even after sarcoidosis was diagnosed, she refused oral corticosteroid therapy and was followed up without treatment. Her chest X‐ray and CT findings were unremarkable, and the serum angiotensin‐converting enzyme (ACE) level fluctuated between 25 and 32 U/mL (Fig. 1A). In early 2014, her skin eruptions became worse, and we decided to treat her skin sarcoidosis with minocycline monotherapy.

Notably, after the initiation of minocycline (11 April 2014), her skin eruptions, which had never improved in >15 years, started resolving gradually. The plaque redness decreased, the bulging started to heal (Fig. 1B), and her serum ACE level also decreased (Fig. 1A). After almost one year of the monotherapy, her skin sarcoid plaques almost disappeared with only slight scarring. Moreover, there was a marked decrease in her serum ACE level (from 24.7 to 13.6 U/L). Despite these significant improvements, the amelioration of pulmonary sarcoidosis was minimal. Over the last two years, the patient has continued to receive the minocycline monotherapy without any significant adverse events.

We retrospectively performed immunohistochemical staining on a skin biopsy (paraffin block specimen obtained on 14 April 1999) using a *P. acnes*‐specific monoclonal antibody (PAB antibody) that reacts with cell‐membrane‐bound lipoteichoic acid 3. Notably, despite the absence of staining with anti‐mycobacterium tuberculosis antibody, the PAB antibody stain was positive (Fig. 2D), revealing the presence of *P. acnes* in the skin sarcoid granulomatous lesion.

## Discussion

The most likely aetiology of sarcoidosis is an allergic reaction to a chronic persistent infection with *P. acnes*, a pathogenic bacterium that causes acne vulgaris. *P. acnes* invades from the external environment through the airway and causes subclinical infection. Sarcoidosis occurs only in individuals with excessive immunoreactions due to the intracellular proliferation of *P. acnes* 1.

In our case, the patient had been followed up for 15 years without any treatment, and the minocycline monotherapy, without corticosteroids or other immunosuppressive agents, resulted in the resolution of skin lesions and decreased serum ACE levels. Moreover, PAB staining of a skin biopsy specimen confirmed the presence of *P. acnes*. This result raises the question of whether the eradication of *P. acnes* might have contributed to the remission. In that case, the minocycline monotherapy can be expected to be as effective for lung lesions as for skin lesions. However, in our patient, the improvement in the lung lesions was much lesser than that in the skin lesions.

The efficacy of antimicrobial agents on sarcoidosis has been reported in several studies. Bachelez et al. have reported the efficacy of minocycline for treating 12 patients with chronic skin sarcoidosis 2. In their study, a 200 mg dose of minocycline was administered to 12 patients with chronic skin sarcoidosis for a median duration of 12 months, and complete and partial responses were observed in eight and two patients, respectively. Moreover, of the seven patients with complete responses who discontinued minocycline, three experienced relapse. Furthermore, in our patient, the effect of the antibiotic on the lung lesions was mostly poor. Minocycline is reportedly effective on ocular sarcoidosis lesions 4. Recently, Takemori et al. have reported the use of clarithromycin to treat *P. acnes* infection of the lymph nodes in patients with acute‐onset sarcoidosis 5. Their study suggested that remission results from clarithromycin‐induced apoptosis through immunosuppression or immunomodulation.

Minocycline—a member of tetracyclines—has various immunomodulating properties; tetracyclines inhibit the migration and chemotaxis of leukocytes 6, whereas minocycline and doxycycline inhibit the activation and proliferation of T cells 7. Additionally, minocycline and doxycycline inhibit granuloma formation 8. Moreover, the growing interest in minocycline has led to detailed evaluations of its therapeutic efficacy in many other experimental disease models, such as inflammatory bowel disease, diabetes, fragile X syndrome, cerebral ischaemia, and human immunodeficiency virus infection 1.

In the present case, it was assumed that the various non‐antimicrobial activities of minocycline, including its immunomodulating activity, contributed to its efficacy on the skin lesions. The differences between its efficacy on skin and pulmonary lesions may also be attributable to the differences in the pathogenic mechanisms between the two organs. The further accumulation of cases and detailed studies are, therefore, warranted to elucidate the mechanism in detail.

### Disclosure Statement

Appropriate written informed consent was obtained for publication of this case report and accompanying images.
